# Experience with Berlin heart support in children with a focus on device removal

**DOI:** 10.1016/j.xjon.2025.09.025

**Published:** 2025-09-25

**Authors:** Valerii Iaprintsev, Igor E. Konstantinov, Edward Buratto, Tyson A. Fricke, Lucas Eastaugh, Christian P. Brizard, Stephanie Perrier, Jacob Mathew

**Affiliations:** a Department of Cardiac Surgery, The Royal Children's Hospital, Melbourne, Australia; b Department of Paediatrics, University of Melbourne, Melbourne, Australia; c Murdoch Children's Research Institute, Melbourne, Australia; d Melbourne Children's Centre for Cardiovascular Genomics and Regenerative Medicine, Melbourne, Australia; e Department of Cardiology, The Royal Children's Hospital, Melbourne, Australia

**Keywords:** Berlin heart, cardiomyopathy, mechanical circulatory support, myocardial recovery, pediatric HF, ventricular assist device

## Abstract

**Objective:**

The Berlin Heart EXCOR is the only durable ventricular assist device available for infants and small children. A subset of patients supported by the Berlin Heart EXCOR device experience cardiac recovery. This study aimed to evaluate the outcomes of Berlin Heart EXCOR support at our center and identify the factors associated with successful device explantation.

**Methods:**

This retrospective observational study included all consecutive patients who underwent Berlin Heart EXCOR implantation at the Royal Children's Hospital from 2009 to 2024.

**Results:**

A total of 72 patients received Berlin Heart EXCOR support during the study period. The median age at implantation was 1.0 year (interquartile range, 0.44-2.92), median body surface area was 0.43 m^2^ (interquartile range, 0.30-0.56), and median support duration was 127 days (interquartile range, 59-219). Heart transplantation was performed in 47 patients (65.3%), and 10 patients (13.9%) achieved device explantation without transplantation—4 with dilated cardiomyopathy and 6 with myocarditis. Fourteen patients (19.4%) died on support, and 1 patient remained supported at analysis. There were no significant differences in age, body surface area at implantation, or duration of support between those who recovered and those who died or underwent transplantation. Of 30 patients aged less than 2 years with body surface area between 0.35 and 0.55 m^2^, 9 achieved recovery. In contrast, none of the 23 patients with body surface area less than 0.35 m^2^ and only 1 of 18 patients with body surface area more than 0.55 m^2^ successfully underwent explantation. In multivariable Cox regression, myocarditis was significantly associated with successful explantation (hazard ratio, 11.6, 95% CI, 0.987-135.1; *P =* .05). Because of the low number of recovery events and near-complete separation by body surface area category, Firth's penalized Cox regression was applied. Within this model, body surface area between 0.35 and 0.55 m^2^ was strongly associated with recovery (hazard ratio, 27.4, 95% CI, 3.46-3539.5; *P =* .0003).

**Conclusions:**

This study demonstrates that a strategy focused on evaluation of myocardial recovery may increase the rate of device removal. The majority of successful device removals occurred in children younger than 2 years of age with a body surface area of 0.35 to 0.55 m^2^. Patients with myocarditis are more likely to have a successful device removal. We believe that every patient must be evaluated for myocardial recovery and device removal before committing the patient to heart transplantation.

## Graphical Abstract

**Figure:**
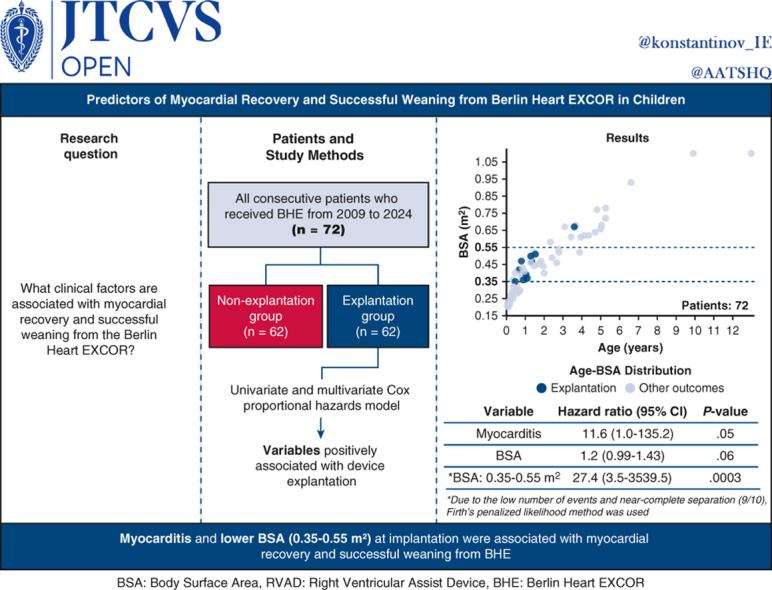

**Figure:**
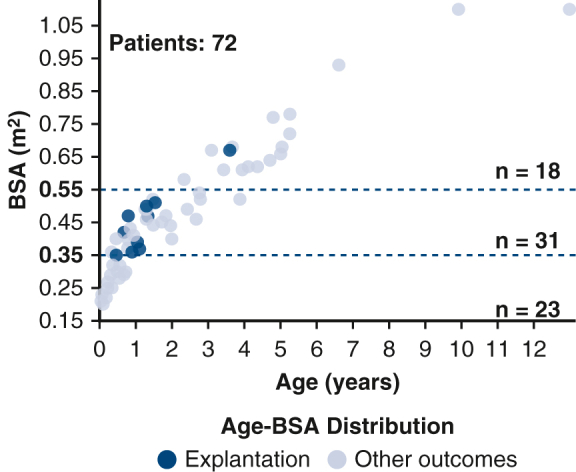
Age–body surface area distribution in patients with a BHE device.

**Central Message**

The rate of BHE removal can be increased with a focused strategy.

**Perspective**

The majority of successful device removal occurred in children younger than 2 years of age with a body surface area of 0.35 to 0.55 m^2^.

For infants and small children with advanced heart failure (HF) unresponsive to medical therapy, the Berlin Heart EXCOR (BHE) is the most widely used durable ventricular assist device (VAD). It is suitable for patients ranging in size from 3 kg to adult weight and serves primarily as a bridge to heart transplantation.^1^ The effectiveness of the BHE in reducing waitlist mortality is well established.^2^^,^^3^ Support duration can range from weeks to more than 1 year, particularly due to the scarcity of suitable donors for very small children.^4^, ^5^, ^6^ Remarkably, in approximately 9% of pediatric patients supported with the BHE, partial or complete myocardial recovery occurs, allowing for successful device explantation and avoidance of transplantation.^7^^,^^8^ Moreover, myocardial recovery during VAD support has been observed not only in cases of myocarditis or postcardiotomy syndrome but also in children with congenital heart disease (CHD) and various cardiomyopathies.

On the other hand, BHE support is associated with a high risk of stroke, with reported rates ranging from 20% to 40%.^4^^,^^9^^,^^10^ Additionally, prolonged BHE support may be linked to increased variability in outcomes and higher overall morbidity and mortality.^8^ Therefore, establishing a prompt pathway to transplantation or recovery is critical in this population. Although our national pediatric heart transplantation program is cost-effective and achieves excellent outcomes,^11^ the geographic isolation of our country contributes to a significant shortage of donor hearts. This, in turn, prolongs BHE support and underscores the need to identify candidates for whom bridge to recovery and device explantation may be feasible. Several factors have been associated with enhanced myocardial recovery, including younger age,^12^ lower body surface area,^13^ shorter duration of VAD support,^14^ and a less severe initial clinical status.^6^ However, the ability to predict which patients will recover remains limited. This study aims to identify predictors of successful myocardial recovery in the smallest patients supported with BHE and to demonstrate a surgical technique that enables safe and effective explantation of the device. A better understanding of the recovery phenomenon and improved predictive capacity may help increase the proportion of patients who can avoid heart transplantation and lifelong immunosuppression.

## Material and Methods

### Patients

**Figure 1:**
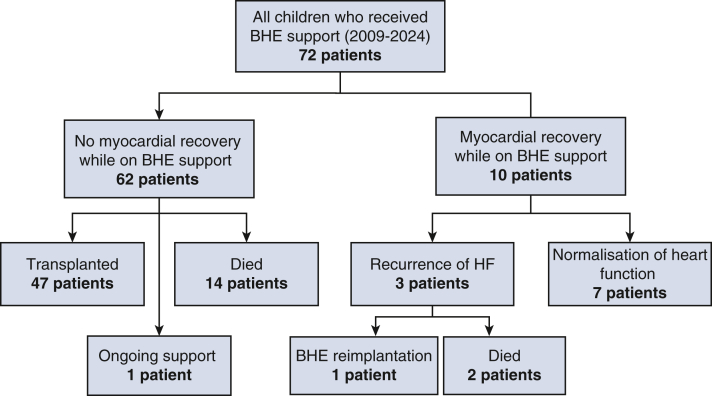
Flow chart of patients undergoing BHE support (2009-2024). *BHE,* Berlin Heart EXCOR.

The study was approved by the Royal Children's Hospital Human Research Ethics Committee HREC/21/QCHQ/80891 on November 11, 2021. All data were collected retrospectively from hospital electronic medical records. All the consecutive patients who underwent BHE (Berlin Heart GmbH) implantation at The Royal Children's Hospital from 2009 to 2024 were identified from the hospital's cardiac surgery database and included in the study (Figure 1).

### Indications for Device Removal

In our hospital, a protocol similar to the one previously described is used.^15^ All patients are assessed for improvement of left ventricular (LV) function within 2 weeks of support. If there is an improvement, the VAD weaning protocol is initiated. If there is not improvement, then assessment is performed every 2 weeks. The initial 2 to 3 weaning studies are performed with noninvasive monitoring on the general cardiology ward. Thirty minutes before the study, bivalirudin infusion is ceased and the patient is typically given a 100 U/kg bolus of heparin. This is repeated as required to maintain an activated clotting time greater than 150 seconds throughout the study. With monitoring of heart rate, blood pressure, central venous, oxygen saturation, and lactate, together with live echocardiographic monitoring, the VAD rate is weaned to a minimum of 5 beats per minute, typically over 20 to 30 minutes. If the patient demonstrates hemodynamic stability and there is no significant burden of fibrin or clot in the circuit, they are maintained at this minimal VAD rate for 60 minutes. A trial is considered successful if the patient maintains hemodynamic stability, as evidenced by a heart rate increment of less than 10 to 20 beats per minute, stable blood pressure, a mixed venous saturation greater than 60%, and normal lactic acid level, together with a left ventricular end-diastolic dimension z-score 2 or less and an ejection fraction 45% to 50% or more in the absence of significant (mild or less) mitral or tricuspid regurgitation. If the initial trials with noninvasive assessment are encouraging, a further trial is conducted under general anesthesia in the cardiac catheterization laboratory, allowing for invasive measurement of cardiac output, wedge pressure, and pulmonary arterial and right atrial pressure. In addition to the parameters noted previously, the invasive trial would be considered favorable if the cardiac index exceeds 2.5 L/min/m^2^, the mean right atrial pressure is less than 10 mm Hg, and the pulmonary capillary wedge pressure is less than 13 mm Hg. Patients who satisfied those explanation criteria listed above typically proceeded with VAD explantation within 48 hours of the trial. Data from weaning trials in recovered patients are presented in Table E1.

### Technique for Device Removal

The explantation of the BHE was performed using cardiopulmonary bypass (CPB) with the heart beating throughout the procedure (Video 1). After bypass initiation, the BHE outflow cannula was dissected, divided between clips, and removed. After the cannula excision, the left ventricle was thoroughly inspected for clots, which were removed if present. The ventriculotomy was closed with a patch sutured to the endocardium at the LV apex in a continuous fashion.

### Management After Device Removal

Patients with a primary cardiomyopathy or those with any degree of systolic dysfunction postexplantation were managed with typical HF therapy, including angiotensin-converting enzyme inhibition and beta-blockade, or in the most recent era, valsartan-sacubitril and dapagliflozin. Because there is a risk of apical thrombus formation after the explantation and over-sewing of the LV apex, patients were maintained on systemic anticoagulation with warfarin or apixaban for 3 months after device removal.

### Statistical Analysis

Data were analyzed using Stata (version 18, StataCorp) and R (R Foundation for Statistical Computing). Continuous variables are presented as medians with interquartile ranges (IQRs), and categorical variables are presented as frequencies with percentages. Comparisons of continuous variables were performed using the Student *t* test (for Gaussian distributions) or the Mann–Whitney test (for non-Gaussian distributions). Categorical variables were compared using the chi-square test or Fisher exact test when cell counts were less than 5.

Kaplan–Meier curves were used to display freedom from study outcomes, with follow-up data censored at death, loss to follow-up, or December 2024, whichever occurred first. For time-dependent end points (eg, death, transplant, recovery, or ongoing VAD support), cumulative incidence curves were generated.

Univariable and multivariable Cox proportional hazards analysis was performed to identify independent risk factors for outcomes. Cox regression with Firth's penalized likelihood was used to assess the association between body surface area (BSA) and myocardial recovery. This approach was chosen near-complete separation of outcomes when comparing recovery rates between patients with BSA between 0.35 and 0.55 m^2^ and those outside this range. The analysis was performed using the “coxphf” package in R.

## Results

### Overall Cohort

Between 2009 and 2024, the BHE was implanted in 72 patients. In 60 patients (83.3%), the BHE was used for LV support (left ventricular assist device [LVAD]), in 3 patients (4.2%) for the right ventricle support (right ventricular assist device), and in 9 patients (12.5%) support of both right and left ventricles was required (biventricular assist device) (Table 1).

**Table 1:** Basic characteristics of the overall cohort of patients supported with Berlin Heart EXCOR

Characteristic	Whole group, n = 72	Not explanted, n = 62	BHE explant, n = 10	*P* value
Age, y, median (IQR)	1.06 (0.44-2.92)	1.09 (0.37-3.42)	1.06 (0.78-1.32)	.99
Weight, kg, median (IQR)	9.3 (5.5-12.8)	9.3 (5.4-13.3)	9.5 (7.8-10.8)	.59
Male, n (%)	38 (52.8)	32 (51.6)	6 (60)	.62
Weight group, n (%)				
<5 kg	14 (19.4)	14 (22.6)	0	--
5-10 kg	31 (43.1)	25 (40.3)	6 (60)	.24
>10 kg	27 (37.5)	23 (37.1)	4 (40)	.86
BSA, m^2^, median (IQR)	0.43 (0.3-0.56)	0.43 (0.29-0.61)	0.45 (0.37-0.5)	.67
Device type, n (%)				
LVAD	60 (83.3)	51 (82.3)	9 (90)	.54
RVAD	3 (4.2)	3 (4.8)	0	--
BiVAD	9 (12.5)	8 (12.9)	1 (10)	.8
Ventilatory support, n (%)	48 (66.7)	39 (62.9)	9 (90)	.09
Previous ECMO, n (%)	33 (45.8)	28 (45.2)	5 (50)	.78
ECMO support d, median (IQR)	7 (4-10)	6.5 (4-9)	7 (6-14)	.28
Previous surgery, n (%)	11 (15.3)	11 (17.7)	0	--
Centrifugal VAD prior BHE, n (%)	15 (20.8)	14 (22.6)	1 (10)	.36
BHE support time, median (IQR)	127 (59-212)	127 (61-210)	126.5 (34-221)	.79
Etiology of heart failure, n (%)				
Dilated cardiomyopathy	44 (61.1)	40 (64.5)	4 (40)	.14
Restrictive cardiomyopathy	2 (2.8)	2 (3.2)	0	--
Myocarditis	15 (20.8)	9 (14.5)	6 (60)	.001
Congenital heart disease	8 (11.1)	8 (12.9)	0	--
Other	3 (4.2)	3 (4.8)	0	--
INTERMACS profile, n (%)				
1	33 (45.8)	28 (45.2)	5 (50)	.703
2	20 (27.8)	17 (27.4)	3 (30)	.97
3	17 (23.6)	15 (24.2)	2 (20)	.94
4	2 (2.8)	2 (3.2)	0	--
Initial treatment strategy, n (%)				
Bridge to transplant	54 (75.0)	51 (82.3)	3 (30)	.001
Bridge to recovery	11 (15.3)	6 (9.7)	5 (50)	.001
Bridge to decision	7 (9.7)	5 (8.1)	2 (20)	.24
Admission to implant d, median (IQR)	9 (5-15.5)	8 (5-15)	14.5 (10-18)	.18

*BHE,* Berlin Heart EXCOR; *IQR,* interquartile range; *BSA,* body surface area; *LVAD,* left ventricular assist device; *RVAD,* right ventricular assist device; *BiVAD,* biventricular assist device; *ECMO,* extracorporeal membrane oxygenation.

**Figure 2:**
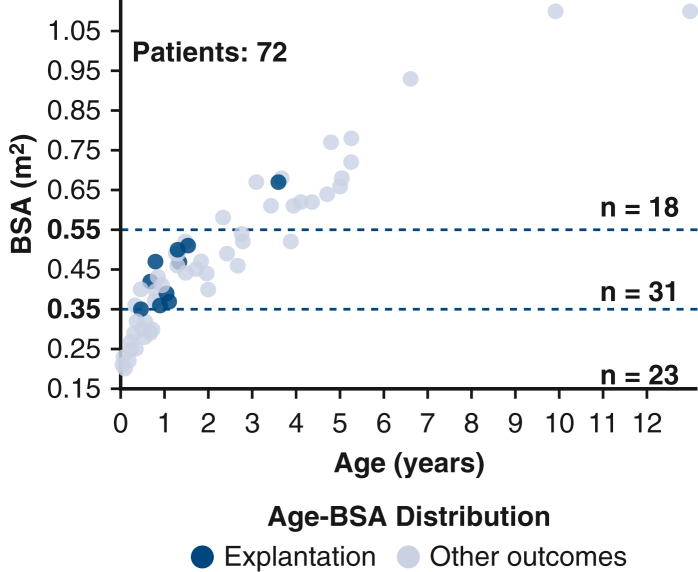
Age–BSA distribution in the overall cohort of patients supported with the BHE, highlighting those with device removal. *BSA,* Body surface area.

The median age at BHE implantation was 0.99 years (IQR, 0.43-2.76 years), with a median weight of 9.3 kg (IQR, 5.5-12.8 kg), median height of 77 cm (IQR, 61.9-93.4 cm), and median BSA of 0.43 m^2^ (IQR, 0.3-0.56 m^2^) (Figure 2). The distribution of patient weights was as follows: A total of 14 patients (19.4%) weighed less than 5 kg, 31 patients (43.1%) weighed between 5.1 and 10 kg, and 27 patients (37.5%) weighed more than 10 kg.

The primary indications for BHE implantation were HF due to dilated cardiomyopathy (DCM) in 44 patients (61.1%), including 9 with LV noncompaction; myocarditis in 15 patients (20.8%), of whom 11 had a confirmed diagnosis and 4 had a probable diagnosis (2 of these patients also had noncompaction); CHD in 8 patients (11.1%); restrictive cardiomyopathy in 2 patients; and other diagnoses in 3 patients (4.2%). Patients with CHD included failed univentricular palliation (n = 5) and congenitally corrected transposition of the great arteries (n = 3). The other diagnoses included isolated noncompaction cardiomyopathy (n = 1), familial arrhythmogenic cardiomyopathy (n = 1), and neonatal myocardial infarction (n = 1).

All proven myocarditis diagnoses in our cohort (11/15, 73.3%) were confirmed by biopsy, demonstrating inflammatory infiltration and necrosis consistent with the Dallas criteria, in addition to clinical evaluation. The remaining 4 cases (26.7%) were classified as probable myocarditis, with histopathology showing inflammatory infiltration but lacking definitive necrosis, along with clinical findings suggestive of myocarditis. However, the specific viral etiology was confirmed in only 6 of 15 cases (40%), with parvovirus identified in 4 children (26.7%) and enterovirus in 2 children (13.3%). Among the 6 patients with myocarditis who recovered, the diagnosis was biopsy proven in 5 (83.3%). Notably, 3 of these recoveries (50%) were associated with parvovirus infection, 1 (16.7%) with either parvovirus or respiratory syncytial virus, and in 2 cases (33.3%) the viral cause remained unknown. Additional information on histopathological findings is provided in Table E2.

The BHE was used as a bridge-to-transplant (BTT) in 54 patients (75.0%), bridge-to-recovery (BTR) in 11 patients (15.3%), and bridge-to-decision in 7 patients (9.7%). The median duration of BHE support was 127 days (range, 1-588 days), with 7 patients (9.7%) receiving support for more than 1 year. According to Interagency Registry for Mechanically Assisted Circulatory Support (INTERMACS) profiles, the cohort included 33 patients (45.8%) in profile 1, 20 patients (27.8%) in profile 2, 17 patients (23.6%) in profile 3, and 2 patients (2.8%) in profile 4.

The median time from hospital admission to BHE implantation was 9 days (IQR, 5-15.5). Extracorporeal membrane oxygenation (ECMO) was required for 33 patients (45.8%) before VAD implantation, with a median ECMO duration of 7 days (range, 0.5-18 days).

### Overall Outcomes

At the time of the study, 47 patients (65.3%) received a heart transplant post-BHE, 10 patients (13.9%) were successfully weaned (4 with DCM and 6 with myocarditis), 14 patients (19.4%) died during therapy, and 1 patient (1.4%) was still receiving VAD support. Causes of death included fatal neurological events in 6 patients (42.9%), multiple organ system failure in 6 patients (42.9%), and cannula breaches in 2 patients (14.2%) (with the duration of VAD support being 308 and 396 days, respectively). Two of the patients who died had univentricular circulation.

**Figure 3:**
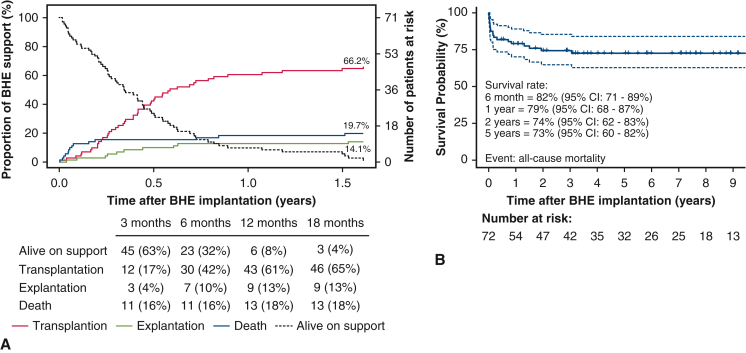
A, Competing outcomes (transplantation, explantation due to recovery, death, and ongoing support) among 72 children supported with the BHE between 2009 and 2024. Estimated cumulative incidence for each outcome is shown at 3, 6, 12, and 18 months of follow-up since device implantation. B, Kaplan–Meier survival curve for long-term survival in the same cohort. Survival estimates were not censored at the time of transplantation or explantation. 95% CI. *BHE,* Berlin Heart EXCOR.

The competing outcome analysis revealed that 30 patients (42%) received a heart transplant within 6 months of BHE placement and 43 patients (61%) received a heart transplant within 1 year (Figure 3, *A*). It was also found that in 7 (10%) of 10 cases of VAD weaning, the explantation was made within 6 months from the date of installation of the VAD, but only 3 patients (4%) were disconnected from the VAD within the first 3 months. The model also demonstrated that the critical period in terms of mortality is the first 3 months, with 11 (16%) of 14 deaths occurring during this period.

The estimated overall survival at 6 months and 1, 2, and 5 years was 82% (95% CI, 71%-89%), 79% (95% CI, 68%-87%), 74% (95% CI, 62%-83%), and 73% (95% CI, 60%-82%), respectively (Figure 3, *B*). This demonstrates stable long-term survival after BHE implantation if a patient survived through the first year after MCS initiation.

### Patients Who Underwent Device Removal

The cohort of patients who recovered during VAD support includes 10 patients (13.9%), of whom 6 are male and 4 are female (Table 1). In 9 of 10 patients, the BHE was used as an LVAD, and in only 1 patient as a biventricular assist device. The reasons for MCS in this group were idiopathic DCM in 4 patients (40%) and myocarditis in 6 patients (60%). Additionally, the recovered cohort did not include patients with CHD and previous cardiac surgeries. Median of admission to MCS device implantations days was 14.5 (IQR, 10-18 days). Preoperatively, 5 patients were in cardiogenic shock (INTERMACS profile 1, 50%), 3 patients were in progressive decline with inotropic support (INTERMACS profile 2, 30%), and 2 patients were relatively stable but dependent on inotropes (INTERMACS profile 3, 20%).

Concomitantly with 5 patients supported by ECMO before BHE implantation, the median duration of ECMO support in this group was 7 days (IQR, 6-14 days). The initial strategy was BTR in 5 patients, BTT in 3 patients, and bridge-to-decision in 2 patients.

The median age at the time of implant among recovered patients was 1.06 years (IQR, 0.78-1.32 years), median weight was 9.5 kg (IQR, 7.8-10.8 kg), and median BSA was 0.45 m^2^ (IQR, 0.37-0.5 m^2^) (Figure 2). Six patients weighed between 5 and 10 kg, 4 patients weighed more than 10 kg, and none weighed less than 5 kg. No recovery was observed among the 23 patients with BSA less than 0.35 m^2^. Only 1 patient with BSA more than 0.55 m^2^ achieved recovery, whereas the remaining 9 recovered patients were within the BSA range of 0.35 to 0.55 m^2^.

The median duration of BHE support in this group was 126.5 days (IQR, 34-221 days). The shortest duration was 14 days, and the longest one was 559 days. Both were observed in patients with DCM. Additionally, in 8 patients 15-mL pumps were used, and in 2 patients 25-mL pumps were used.

None of the patients in the recovered subgroup had a BSA of less than 0.35 m^2^. However, the collected data did not show a significant distinction between patients with a BSA of less than 0.35 m^2^ (n = 23) and the rest of the cohort, except for the primary outcomes, where death was significantly more frequent in the smaller BSA group (12.2% vs 34.8%, *P =* .02). Furthermore, 9 of 10 recovered patients were aged 6 to 24 months. When comparing patients in this age group with the rest of the cohort (30 vs 42 patients, respectively), the analysis showed that left-sided VADs were more predominant in this age group (76.2% vs 93.3%, *P =* .05). Additionally, this group did not include any patients with CHD, whereas the rest of the cohort included 8 patients with CHD. It also had fewer critically ill patients, as indicated by the lower proportion of those with an INTERMACS 1 profile (54.8% vs 30.0%, *P =* .04).

**Figure E1:**
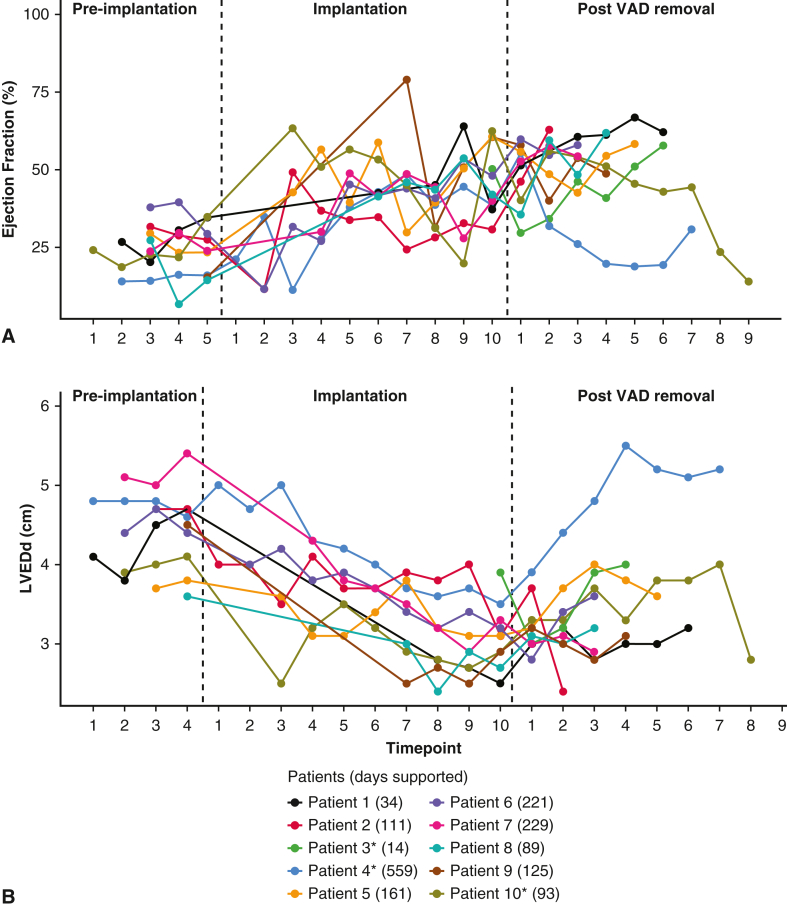
Ejection fraction (A) and LVEDd (B) in patients who recovered after BHE support. All available echocardiograms are plotted in sequential order per patient and grouped into 3 equal phases: preimplantation, implantation, and postimplantation. This time-normalized approach allows comparison across patients with variable VAD durations. Asterisks (∗) indicate postexplant HF relapse. *LVEDd,* Left ventricular end-diastolic dimension; *VAD,* ventricular assist device.

At the time of analysis, 7 patients (70%) were doing well. Two patients (20%) died due to a relapse of HF symptoms 45 and 151 days after explantation, and 1 patient (10%) required VAD support again 87 days after explantation. Patient 1 was weaned off the device after 14 days of support and experienced a complex postexplant course, including chylothorax requiring thoracic duct ligation, pneumonia (methicillin-resistant *Staphylococcus aureus*), and subdural hemorrhage. These complications contributed to recurrent HF. Because of neurological complications, the parents decided not to pursue reimplantation or transplantation. Patient 2 was weaned off after 559 days of support after recovery. The patient had developed short bowel syndrome due to prior bowel resection for necrotizing enterocolitis, making him ineligible for transplantation. The improved LV function allowed explantation of the VAD. The patient relapsed 151 days after explantation. Because the patient was not eligible for heart transplantation, he was not eligible for implantation of VAS as a BTR. Patient 3, who was weaned from BHE after 93 days of support, carried a variant of uncertain significance in the *LRPPRC* gene, associated with mitochondrial cardiomyopathy. Although evidence is limited, this may have contributed to an unfavorable myocardial recovery trajectory despite initial improvement. The VAD was reimplanted as a BTR. Additionally, all relapsed patients had an initial diagnosis of DCM that led to BHE implantation, whereas 6 of 7 patients who were doing well had a diagnosis of myocarditis. More detailed information about these patients is provided in Table E3, and changes in echocardiographic parameters are shown in Figure E1.

### Factors Affecting Device Removal

Univariable Cox proportional hazards analysis (Table E4) for primary outcomes showed that the recovery outcome is affected favorably by the diagnosis of myocarditis (hazard ratio [HR], 7.9, 95% CI, 1.97-31.62, *P =* .003) and initial BTR strategy (HR, 5.95, 95% CI, 1.59-22.29, *P =* .008). Multivariable analysis (Table 2) demonstrated that only myocarditis remained a factor associated with successful device removal (HR, 11.6, 95% CI, 0.99-135.2, *P =* .05). Age at implantation (HR, 0.79, 95% CI, 0.61-1.01, *P =* .06) and BSA (HR, 1.11, 95% CI, 0.99-1.43, *P =* .06) did not appear to affect the outcome on multivariable analysis, likely due to small numbers. However, when analyzed together, age at implantation (HR, 0.77, 95% CI, 0.63-0.94, *P =* .01) and BSA (HR, 6.83, 95% CI, 1.72-27.1, *P =* .006) became significant, likely due to strong intervariable correlation. Interestingly, the BTR strategy lost its significance in the multivariable model (HR, 0.15, 95% CI, 0.01-2.82, *P =* .2). Additionally, sex and previous use of ECMO did not affect the recovery status in the univariable model or multivariable model.

**Table 2:** Multivariable Cox proportional hazards analysis for primary outcomes (mortality, transplant, and recovery) in patients who underwent Berlin Heart EXCOR implantation

Variable	Mortality		Transplant		Recovery	
	Hazard ratio (95% CI)	*P* value	Hazard ratio (95% CI)	*P* value	Hazard ratio (95% CI)	*P* value
Male sex	0.49 (0.14-1.75)	.27	1.37 (0.65-2.86)	.41	1.10 (0.27-4.44)	.89
Age at implant, mo	1.09 (1.01-1.18)	.03	0.99 (0.94-1.04)	.64	0.79 (0.61-1.01)	.06
BSA, m^2^	0.89 (0.80-0.99)	.03	1.02 (0.97-1.08)	.46	1.19 (0.99-1.43)	.06
Previous ECMO	1.81 (0.47-6.93)	.39	0.83 (0.43-1.61)	.59	1.96 (0.48-7.99)	.35
Myocarditis	5.64 (0.94-33.69)	.06	0.96 (0.13-7.26)	.97	11.55 (0.99-135.18)	.05
CHD	2.72 (0.17-43.58)	.48	0.88 (0.09-9.09)	.92	-	-
Previous surgery	2.03 (0.21-19.49)	.54	0.65 (0.86-4.87)	.67	-	-
BTR strategy	0.38 (0.05-2.87)	.35	0.39 (0.04-3.74)	.41	0.15 (0.01-2.82)	.20

*BSA,* Body surface area; *ECMO,* extracorporeal membrane oxygenation; *CHD,* congenital heart disease; *BTR,* bridge-to-recovery.

Our cohort included a substantial proportion of patients with very low BSA values. Specifically, 54 patients (75.0%) had a BSA below 0.55 m^2^, and 23 patients (32%) had a BSA below 0.35 m^2^. Notably, as shown in Figure 2, no patient with a BSA below 0.35 m^2^ achieved myocardial recovery. These findings suggest that the Cox model may reflect a positive association between BSA and recovery for patients closer to the 0.55 m^2^ mark. Furthermore, we identified the range of 0.35 to 0.55 m^2^ as potentially optimal for recovery, because 9 of 10 recovered patients were within this interval at the time of VAD implantation. It is possible that the patients who present earliest have the most severe phenotype, whereas the older patients have less regenerative capacity. Thus, we can hypothesize that patients in the age range of 6 months to 2 years and BSA 0.35 to 0.55 m^2^ represent the optimal intersection of regenerative capacity and severity of cardiomyopathy. The strong association observed between BSA 0.35 and 0.55 m^2^ and myocardial recovery was demonstrated as statistically significant (HR, 27.4, 95% CI, 3.46-3539.5, *P =* .0003) with Firth penalization, which was implemented due to the low number of events and near perfect separation, suggesting this range may be optimal for favorable outcomes. However, the wide CI reflects the small number of recovery events and should be interpreted with caution.

## Discussion

The current study demonstrated that BHE placement can yield favorable results reaching successful outcomes in 79% of patients, serving as an effective bridge to heart transplantation in young patients despite relatively long duration of support. The heart function recovery and BHE weaning rate in our cohort was 14%, which is higher than what has been reported by other groups, which was reported to be 8% to 9%.^7^^,^^8^

To investigate the factors contributing to myocardial recovery during BHE therapy, we compared demographic and clinical characteristics between patients who were successfully weaned from the VAD and those who were not. Interestingly, no significant differences were observed in the characteristics listed in Table 1, except for the initial choice of support strategy (eg, BTR or BTT). However, among the group of patients whose cardiac function was restored during BHE therapy, none weighed less than 5 kg, whereas in the nonrecovery group, 14 patients (19.4% of the total cohort) were under this weight. This finding is intriguing because the prevailing theory suggests that younger age and, as such, lower patient weight may facilitate myocardial recovery.^13^ This interaction implies that the positive effect of BSA on myocardial recovery diminishes with increasing age. One possible explanation is that higher BSA could indicate greater cardiac reserve and metabolic capacity in younger patients, which may enhance recovery potential, whereas older age is often associated with reduced regenerative capacity.

Furthermore, we identified the range of 0.35 to 0.55 m^2^ as potentially optimal for recovery, because 9 of 10 recovered patients were within this interval at the time of VAD implantation. It is possible that the patients who present earliest have the most severe phenotype, whereas the older patients have less regenerative capacity. Thus, we can hypothesize that patients in the age range of 6 months to 2 years and BSA 0.35 to 0.55 m^2^ represent the optimal intersection of regenerative capacity and severity of cardiomyopathy.

One may think that a possible explanation for the absence of recovery in the less than 0.35 m^2^ group might be the lower prevalence of myocarditis. However, in our cohort, myocarditis was diagnosed in 5 of 23 patients (22%) with BSA less than 0.35 m^2^ and in 10 of 31 patients (32.3%) with BSA between 0.35 and 0.55 m^2^. This difference was not statistically significant (*P =* .55), suggesting that the disparity in recovery is unlikely to be explained by myocarditis status alone.

It is worth noting that only 3 of the 10 recovered patients were weaned off within the first 3 months of support, with 7 of 10 being weaned within the first 6 months. This contrasts with findings from other groups, which suggest that myocardial recovery and VAD weaning typically occur during the first 3 to 4 months of support.^1^^,^^12^^,^^16^ Additionally, we observed an outlier in our cohort who was successfully weaned after 559 days of VAD support. This finding challenges the theory that prolonged VAD support leads to myocardial atrophy, making recovery unlikely and support studies that demonstrated that cardiomyocytes atrophy and degeneration do not occur even with long-term support.^17^^,^^18^

The initial support strategy (eg, BTR or BTT) did not significantly affect the likelihood of VAD removal. This suggests that the initial clinical impression may not always predict the ultimate outcome. Therefore, clinicians should continuously assess myocardial function, regardless of the initial diagnosis or clinical status, because recovery may still be possible in cases where it seems unlikely at first. The need for technicians and cardiologists to systematically evaluate signs of myocardial recovery in patients on BHE has been emphasized, because early detection may lead to better clinical outcomes.^19^

Although myocardial recovery on MCS is more likely when HF is due to myocarditis or during the postcardiotomy phase, recovery is generally not anticipated in cases of genetically confirmed DCM. For example, Felkin and colleagues^20^ reported myocardial function restoration in 6 of 10 cases of DCM associated with genetic mutation. Our cohort also demonstrated myocardial function recovery not only in patients with myocarditis but also in 4 patients with idiopathic DCM.

Among 10 patients in whom the device was removed, 7 maintained a stable clinical status, and the remaining 3 developed a recurrence of HF. Interestingly, all patients with recurrent HF had an initial diagnosis of idiopathic DCM, which was the primary indication for VAD implantation.

Hetzer and colleagues^21^ reported that only 7 of 23 patients with idiopathic DCM experienced HF recurrence after VAD removal. Among them, 6 successfully underwent transplantation, and 1 died on the waiting list. Likewise, Dandel and colleagues^22^ found that 10 of 32 patients (31.3%) with idiopathic DCM developed recurrent HF after device explantation, with 8 receiving transplants and 2 experiencing cardiac death. Additionally, the RESTAGE-HF study demonstrated that freedom from LVAD reimplantation or transplantation may be as high as 90% at 1 year and 77% at both 2 and 3 years after VAD removal in patients with nonischemic cardiomyopathy.^23^ Despite the relatively high recurrence rate of HF in this group, it rarely leads to mortality, as evidenced by the outcomes reported in both the studies by Hetzer and colleagues and Dandel and colleagues. Furthermore, 5- and 10-year survivals for these patients were 88.4% and 85.8%, respectively,^24^ whereas Miera and colleagues^12^ reported a 10-year survival of 84%. These findings suggest that the VAD explantation in patients with DCM should not be disregarded, although careful follow-up is required of these patients.

Similarly to other reports,^10^^,^^25^ in our cohort, 11 of 14 (79%) deaths occurred within the first 3 months, and the median duration of support was 127 days (IQR, 59-212 days). However, for patients who survived this critical period of 3 months, the survivals were encouraging, showing minimal decline over time, namely, 79%, 74%, and 73% at 1, 2, and 5 years post-BHE implantation, respectively.

Overall, our findings emphasize the importance of continuous myocardial evaluation in patients with BHE support, particularly in those with a BSA between 0.35 and 0.55 m^2^. This group may have an enhanced potential for recovery, enabling VAD removal.

### Limitations

This study has a limitation regarding data acquisition inherent to its retrospective design. Over the 15-year study period, clinical management approaches have evolved, potentially influencing treatment outcomes in this cohort. Additionally, our study is limited by the small number of recovery events, resulting in wide CIs and statistical uncertainty. In addition, BSA was categorized to reflect its nonlinear association with recovery, which may reduce precision. Finally, examining 3 primary outcomes separately introduces a risk of type I error due to multiple testing.

## Conclusions

This study demonstrates that strategy focused on evaluation of myocardial recovery may increase the rate of device removal. The majority of successful device removals occurred in children younger than 2 years of age with a BSA of 0.35 to 0.55 m^2^. Patients with myocarditis are more likely to have a successfully device removal. We believe that every patient must be evaluated for myocardial recovery and device removal before committing the patient to heart transplantation.

## Conflict of Interest Statement

Dr Brizard discloses a financial relationship with Admedus. All other authors reported no conflicts of interest.

The *Journal* policy requires editors and reviewers to disclose conflicts of interest and to decline handling or reviewing manuscripts for which they may have a conflict of interest. The editors and reviewers of this article have no conflicts of interest.

## Figures and Tables

**Table E1:** Catheterization and echocardiographic data collected during weaning trials in patients who recovered after Berlin Heart EXCOR support

Patient 1	**30 bpm 15 min**: No change in ventricular dimensions or function; **15 bpm 15 min**: No change in ventricular dimensions or function			
Patient 2	**60 bpm**: LVEF 47%, LVEDV 38 mL; **30 bpm**: LVEF 36%, LVEDV 36 mL			
Patient 3	**6 bpm**: LVEDd 3.9 cm, LVESd 2.9 cm, FS 25.1%, EF 50.3%			
Patient 4	**60 bpm**: BP 82/38 mm Hg (56), CO 3.07 L/min, CI 3.7 L/min/m^2^, mPAP 17 mm Hg, LVEDP 9 mm Hg, PCWP 9 mm Hg	**30 bpm**: CO 3.37 L/min, CI 4.09 L/min/m^2^, mPAP 19 mm Hg, LVEDP 14 mm Hg, PCWP 11 mm Hg	**6 bpm**: BP 81/46 mm Hg (62), PA Sat 72%, CO 3.23 L/min, CI 3.93 L/min/m^2^, mPAP 24 mm Hg, LVEDP 16 mm Hg, PCWP 14 mm Hg	**Dobutamine:** BP 105/56 mm Hg (78), PA Sat 75%, CO 4.5 L/min, CI 5.47 L/min/m^2^, mPAP 28 mm Hg, LVEDP 15 mm Hg, PCWP 12 mm Hg
Patient 5	**60 bpm**: HR, BP, PA Sat unchanged, LVEDd 3 cm, LVESd 2.3 cm, FS 23%	**30 bpm**: HR, BP, PA Sat unchanged	**6 bpm**: Bullet EF 44%, LVEDd 3.3 cm, LVESd 2.4 cm, FS 27%	
Patient 6	**60 bpm:** BP 51/33 mm Hg (37), PA Sat 73%, TDCO 2.17 L/min, TDCI 4.05 L/min/m^2^, TPG 8 mm Hg, LVEDd 2.7 cm, LVESd 2.2 cm, FS 21%	**30 bpm:** BP 50/32 mm Hg (36), PA Sat 78%, TDCO 2.37 L/min, TDCI 4.42 L/min/m^2^, TPG 8 mm Hg, LVEDd 3.3 cm, LVESd 2.5 cm, FS 24%	**6 bpm:** BP 59/34 (46), PA Sat 77%, TDCO 2.2 L/min, TDCI 4.11 L/min/m^2^, TPG 6 mm Hg, LVEDd 3.3 cm, LVESd 2.7 cm, FS 18%	**Dobutamine:** BP 79/41 mm Hg (57), PA Sat 77%, TDCO 2.57 L/min, TDCI 4.79 L/min/m^2^, TPG 4 mm Hg, LVEDd 3.5 cm, LVESd 2.5 cm, FS 29%
Patient 7	**60 bpm:** BP 114/64 mm Hg (87), PA Sat 71%, TDCO 2 L/min, TDCI 4 L/min/m^2^, TPG 11 mm Hg, LVEDd 3.3 cm, LVESd 2.7 cm, FS 20%	**30 bpm:** PA Sat 75%, TDCO 1.9 L/min, TDCI 3.8 L/min/m^2^, TPG 13 mm Hg, LVEDd 3.4 cm, LVESd 2.7 cm, FS 20%	**6 bpm:** PA Sat 75%, TDCO 1.7 L/min, TDCI 3.4 L/min/m^2^, TPG 12 mm Hg, LVEDd 3.3 cm, LVESd 2.4 cm, FS 27%	**Dobutamine:** BP 120/58 mm Hg (84), PA Sat 74%, TDCO 1.83 L/min, TDCI 3.7 L/min/m^2^, TPG 16 mm Hg, LVEDd 3.3 cm, LVESd 2.2 cm, FS 33%
Patient 8	**60 bpm:** HR 110, BP 69/57 mm Hg (63), EF 51%, MVO2 78.2 mL/min/m^2^, mPAP 17 mm Hg, PCWP 5 mm Hg, PA Sat 79%, CO 1.23 L/min, TDCI 2.53 L/min/m^2^, LVEDd 2.8 cm, LVESd 2.1 cm, FS 23%	**5 bpm:** HR 106, BP 76/52 mm Hg (54), EF 41%, MVO2 65.5 mL/min/m^2^, mPAP 20 mm Hg, PCWP 9 mm Hg, PA Sat 70%, CO 1.44 L/min, TDCI 2.94 L/min/m^2^, LVEDd 2.5 cm, LVESd 2 cm, FS 19%	**5 bpm 30 min:** HR 107, BP 65/36 mm Hg (46), EF 48%, mPAP 24 mm Hg, PCWP 10 mm Hg, PA Sat 66%, TDCI 2.72 L/min/m^2^, CO 1.33 L/min, LVEDd 2.7 cm, LVESd 2.1 cm, FS 23%	**5 bpm 60 min:** HR 102, BP 86/53 mm Hg (65), EF 44%, MVO2 69 mL/min/m^2^, mPAP 19 mm Hg, PCWP 10 mm Hg, PA Sat 69%, TDCI 2.81 L/min/m^2^, CO 1.37 L/min, LVEDd 2.7 cm, LVESd 2.1 cm, FS 20%
Patient 9	**60 bpm**: HR 120, BP 72/46 mm Hg (55), EF 52%, MVO2 65 mL/min/m^2^, mPAP 17 mm Hg, PCWP 10 mm Hg, PA Sat 66%, Fick CI 4.5 L/min/m^2^, TDCI 2.67 L/min/m^2^	**5 bpm:** HR 125, BP 72/38 mm Hg (50), EF 57%, MVO2 66 mL/min/m^2^, mPAP 22 mm Hg, PCWP 12 mm Hg, PA Sat 66%, Fick CI 4.4 L/min/m^2^	**5 bpm 30 min:** HR 124, BP 70/36 mm Hg (49), EF 56%, MVO2 64 mL/min/m^2^, mPAP 22 mm Hg, PCWP 12 mm Hg, PA Sat 66%, Fick CI 4.2 L/min/m^2^	**5 bpm 60 min:** HR 118, BP 75/38 mm Hg (51), EF 56%, MVO2 64 mL/min/m^2^, mPAP 19 mm Hg, PCWP 11 mm Hg, PA Sat 64%, Fick CI 4.4, TDCI 2.33 L/min/m^2^
Patient 10	**85 bpm:** HR 85, BP 61/39 mm Hg (48), EF 53%, MVO2 78.6 mL/min/m^2^, mPAP 14 mm Hg, PCWP 9 mm Hg, PA Sat 72%, Fick CI 3.12 L/min/m^2^, TDCI 1.7 L/min/m^2^	**5 bpm:** HR 96, BP 73/41 mm Hg (49), EF 51%, MVO2 72.7 mL/min/m^2^, mPAP 20 mm Hg, PCWP 9 mm Hg, PA Sat 74%	**5 bpm 30 min:** HR 94, BP 77/43 mm Hg (53), EF 66%, mPAP 16 mm Hg, PCWP 10 mm Hg, PA Sat 68%	**5 bpm 60 min:** HR 99, BP 78/42 mm Hg (52), EF 60%, mPAP 17 mm Hg, PCWP 10 mm Hg, PA Sat 66%, Fick CI 2.82 L/min/m^2^, TDCI 1.53 L/min/m^2^

*bpm,* Beats per minute; *LVEDd,* left ventricular end-diastolic diameter; *LVESd,* left ventricular end-systolic diameter; *EF,* ejection fraction; *BP,* blood pressure; *CO,* cardiac output; *CI,* cardiac index; *mPAP,* mean pulmonary artery pressure; *LVEDP,* left ventricular end-diastolic pressure; *PCWP,* pulmonary capillary wedge pressure; *PA,* pulmonary artery; *Sat,* saturation; *HR,* heart rate; *FS,* fractional shortening; *TDCO,* thermodilution cardiac output; *TDCI,* thermodilution cardiac index; *TPG,* transpulmonary gradient; *MVO2,* myocardial oxygen consumption.

**Table E2:** Histopathological findings from left ventricular core myocardial samples collected at the time of Berlin Heart EXCOR implantation

Patient	Diagnosis	Features	Conclusion	Outcome
Myocarditis				
1	Myocarditis	Lymphocytic infiltrate, myocyte shrinkage, hypereosinophilia, interstitial edema	Myocarditis	Explantation
2	Myocarditis	Myocyte necrosis, nuclear debris, lymphocyte and macrophage infiltrate	Myocarditis	Death
3	Myocarditis	Myocyte necrosis, lymphocytic infiltrate, cell swelling and vacuolation	Myocarditis	Death
4	Myocarditis	Myocyte hypertrophy, fiber disarray, fibrosis, lymphocytic infiltrate	Myocarditis (probable)	Explantation
5	Myocarditis	Fibrotic thickening of the endocardium, lymphocytic infiltrate, hypereosinophilia, granulation tissue	Myocarditis (probable)	Transplantation
6	Myocarditis	Foci of hemopoiesis, myocyte necrosis, lymphocytic infiltrate	Myocarditis	Death
7	Myocarditis	Prominent lymphocytic infiltration, interstitial edema, fibrosis, myocyte shrinkage	Myocarditis (probable)	Transplantation
8	Myocarditis	Interstitial edema, lymphocytic infiltrate, T lymphocytes	Myocarditis (probable)	Death
9	Myocarditis	Interstitial edema, cytoplasmic vacuolation of myocytes, lymphocytic infiltrate, myocyte necrosis	Myocarditis	Transplantation
10	Myocarditis	Interstitial edema, myocyte necrosis, lymphocytic infiltrate	Myocarditis	Explantation
11	Myocarditis	Lymphocytic infiltrate, T lymphocytes, myocyte necrosis, myocyte hypertrophy	Myocarditis	Transplantation
12	Myocarditis	Myocyte hypertrophy, plasma cell infiltrate, interstitial edema, lymphocytic infiltrate	Myocarditis	Explantation
13	Myocarditis	Lymphocytic infiltrate, lymphoid aggregates, hypereosinophilia, cytoplasmic vacuolation of myocytes	Myocarditis	Explantation
14	Myocarditis	Lymphocytic infiltrate, myocyte necrosis, interstitial edema, myocyte hypertrophy	Myocarditis	Transplantation
15	Myocarditis	Lymphocytic infiltrate, interstitial edema, myocyte necrosis	Myocarditis	Explantation
Cardiomyopathy				
16	DCM	Neutrophilic infiltrate, myocyte hypertrophy	Cardiomyopathy	Death
17	DCM	Interstitial edema	No significant abnormality	Transplantation
18	DCM	Areas with loss of myocyte, granulation tissue, hyperchromatic and enlarged nuclei	Hypertrophy	Transplantation
19	DCM	Myofiber disarray, immature pale basophilic matrix between myocytes, fibrosis, sparse inflammatory infiltrate	Cardiomyopathy	Transplantation
20	DCM	Regular arrangement of myocytes	No abnormality	Transplantation
21	DCM	Fibrosis, enlarged and irregular myocyte nuclei	Consistent with DCM	Transplantation
23	DCM	Sparse lymphocytic infiltrate	No histologic abnormality	Transplantation
23	DCM	Neutrophilic infiltrate, regular arrangement of myocytes	Non-specific inflammation	Transplantation
24	DCM	Myocyte hypertrophy, fibrosis, hyperchromatic and enlarged nuclei	Cardiomyopathy	Transplantation
25	DCM	Fibrosis, sparse infiltrate of chronic inflammatory cells, hemorrhage and hemosiderin deposition, regular arrangement of myocytes	Focal fibrosis	Transplantation
26	DCM	Neutrophilic infiltrate, myocyte necrosis, areas of crushed myocardium (zone of ECMO cannula)	Myocarditis	Transplantation
27	DCM	Regular arrangement of myocytes	No histologic abnormality	Transplantation
28	DCM	Regular arrangement of myocytes	No histologic abnormality	Transplantation
29	DCM	Regular arrangement of myocytes	No histologic abnormality	Transplantation
30	DCM	Myocyte hypertrophy	Hypertrophy	Transplantation
31	DCM	Regular arrangement of myocytes, thickening of the endocardium	Endocardial fibroelastosis	Transplantation
32	DCM	Myocyte hypertrophy, interstitial pale basophilic myxoid change, fibrosis, dystrophic calcification	Consistent with DCM	Transplantation
33	DCM	Fibrosis, nuclear enlargement and fiber disarray, myocyte necrosis, dystrophic calcification	Consistent with DCM	Transplantation
34	DCM	Regular arrangement of myocytes, myocyte nuclei size irregularity, interstitial edema	Focal oedema	Transplantation
35	DCM	Mature sparsely cellular collagen, neutrophilic infiltrate, myocyte hypertrophy, myocyte necrosis	Consistent with DCM	Death
36	DCM	Myocyte hypertrophy, fibrosis	Cardiomyopathy	Transplantation
37	DCM	Myocyte hypertrophy	Hypertrophy	Explantation (relapse: death)
38	DCM	Subendocardial fibrosis, focus of calcification	Subendocardial fibrosis	Transplantation
39	DCM	Mild nuclear enlargement, fibrosis, granulation tissue	Cardiomyopathy	Explantation (relapse: death)
40	DCM	Regular arrangement of myocytes	No abnormality	Death
41	DCM	Fibrotic thickening of the endocardium	Endocardial fibroelastosis	Death
42	DCM	Fibrotic thickening of the endocardium	Endocardial fibroelastosis	Transplantation
43	DCM	Fibrotic thickening of the endocardium, cytoplasmic vacuolation of myocytes	Endocardial fibroelastosis	Death
44	DCM	Endocardial fibroelastosis, myocyte hypertrophy, fibrosis, lymphocytic infiltrate	Consistent with DCM	Transplantation
45	DCM	Extensive endocardial fibroelastosis, fibrosis	Consistent with DCM	Transplantation
46	DCM	Myocyte hypertrophy, fibrosis	Interstitial fibrosis	Transplantation
47	DCM	Regular arrangement of myocytes	No abnormality	Transplantation
48	DCM	Myocyte hypertrophy	Hypertrophy	Transplantation
49	DCM	Enlarged hyperchromatic myocyte nuclei, interstitial edema	Cardiomyopathy	Transplantation
50	DCM	Fibrosis, myocyte hypertrophy	Hypertrophy	Transplantation
51	DCM	Disorganization of myocyte architecture, myocyte hypertrophy, enlarged hyperchromatic myocyte nuclei	Cardiomyopathy	Transplantation
52	DCM	Fiber disarray, fibrosis, myocyte hypertrophy	Cardiomyopathy	Transplantation
53	DCM	Diffuse myocyte enlargement, nuclear enlargement and hyperchromasia, fibrosis	Cardiomyopathy	Transplantation
54	DCM	Myocyte necrosis, lymphocytic infiltrate	Myocarditis	Death
55	DCM	Endocardial fibroelastosis, fibrosis	Endocardial fibroelastosis	Transplantation
56	DCM	Mild increase in myocyte nuclear size, endocardial fibroelastosis	Endocardial fibroelastosis	Transplantation
57	DCM	Endocardial fibroelastosis, elastic and collagen fibers in concentric arrangement, glycogen in patchy distribution	Endocardial fibroelastosis	Alive on support
58	DCM	Regular arrangement of myocytes	No abnormality	Explantation
59	DCM	Myocyte hypertrophy, cytoplasmic vacuolation of myocytes, fibrosis	Hypertrophy	Explantation (relapse:VAD)
60	RCM	Regular arrangement of myocytes	No abnormality	Death
61	RCM	Myofiber enlargement and disarray, nuclear enlargement and hyperchromasia	Cardiomyopathy	Transplantation
62	LVNC	Thickened endocardium, trabeculations with staghorn-shaped recesses, endocardial fibroelastosis, focal acute ischemic change	Consistent with left ventricular noncompaction	Transplantation
63	ACM	Myocyte hypertrophy, fibrosis, endocardial fibroelastosis	Cardiomyopathy	Transplantation
Congenital heart disease				
64	CHD	Not available	Not available	Transplantation
65	CHD	Not available	Not available	Death
66	CHD	Not available	Not available	Death
67	CHD	Not available	Not available	Death
68	CHD	Neutrophilic infiltrate, pericardial fibrosis	Pericardial fibrosis	Transplantation
69	CHD	Myocyte hypertrophy, granulation tissue, myocyte necrosis	Myocardial infarction	Transplantation
70	CHD	Not available	Not available	Transplantation
71	CHD	Not available	Not available	Transplantation
72	LV infarct	Not available	Not available	Transplantation

*DCM,* dilated cardiomyopathy; *ECMO,* extracorporeal membrane oxygenation; *RCM,* restrictive cardiomyopathy; *LVNC,* left ventricular noncompaction; *ACM,* arrhythmogenic cardiomyopathy; *VAD*, ventricular assist device.

**Table E3:** Characteristic of 10 patients who underwent Berlin Heart EXCOR explantation for myocardial recovery

Patient no	Sex	Age,y	Weight, kg	Height, cm	BSA, m^2^	Diagnosis	INTERMACS profile	Mechanical ventilation	Previous ECMO	ECMO support, d	Support strategy	LVAD	BiVAD	Pump size	BHE support, d	Status
1	Male	1.32	9.8	81.0	0.47	Myocarditis, proven, unspecified virus	2	1	0		BTR	1	0	25 mL	34	Alive
2	Male	1.09	7.8	70.0	0.37	Myocarditis, probable, unspecified virus	3	0	0		BTD	1	0	15 mL	111	Alive
3	Female	0.45	7.8	65.0	0.35	Idiopathic DCM	1	1	1	7	BTT	1	0	15 mL	14	Dead
4	Male	3.59	15.5	102.0	0.67	Idiopathic DCM	2	1	0		BTD	1	0	25 mL	559	Dead
5	Female	0.66	8.1	72.0	0.42	Myocarditis, proven, parvovirus	2	1	0		BTR	1	0	15 mL	161	Alive
6	Male	1.29	11.0	84.0	0.50	Myocarditis, proven, parvovirus	1	1	1	3	BTR	1	0	15 mL	221	Alive
7	Female	1.03	10.6	77.0	0.39	Myocarditis, proven, parvovirus	3	1	0		BTR	1	0	15 mL	229	Alive
8	Male	0.78	9.2	74.0	0.47	Myocarditis, proven, parvovirus	1	1	1	18	BTR	0	1	15 mL	89	Alive
9	Female	0.88	7.0	71.0	0.36	Idiopathic DCM	1	1	1	6	BTT	1	0	15 mL	125	Alive
10	Male	1.52	10.8	86.0	0.51	Idiopathic DCM	1	1	1	14	BTT	1	0	15 mL	93	reVAD

*BSA,* Body surface area; *ECMO,* extracorporeal membrane oxygenation; *LVAD,* left ventricular assist device; *BiVAD,* biventricular assist device; *BHE,* Berlin Heart EXCOR; *BTR,* bridge-to-recovery; *BTD,* bridge-to-decision; *DCM,* dilated cardiomyopathy; *BTT,* bridge-to-transplant; *reVAD,* ventricular assist device reimplantation.

**Table E4:** Univariable Cox proportional hazard regression models for mortality, transplantation, and recovery

Variable	Mortality		Transplantation		Recovery	
	Hazard ratio (95% CI)	*P* value	Hazard ratio (95% CI)	*P* value	Hazard ratio (95% CI)	*P* value
Male sex	0.67 (0.22-2.01)	.48	1.15 (0.64-2.05)	.64	0.75 (0.21-2.64)	.65
Age at implant, mo	1.01 (0.99-1.03)	.64	1.00 (0.99-1.02)	.61	0.96 (0.90-1.02)	.17
BSA, m^2^	0.99 (0.97-1.02)	.73	1.00 (0.99-1.02)	.69	0.99 (0.95-1.03)	.64
Previous ECMO	3.10 (0.96-9.96)	.06	0.79 (0.43-1.43)	.43	1.39 (0.40-4.81)	.60
Myocarditis	1.75 (0.54-5.67)	.36	0.49 (0.19-1.25)	.14	7.90 (1.97-31.62)	.003
CHD	2.12 (0.57-7.82)	.26	0.84 (0.30-2.36)	.74	-	-
Previous surgery	2.34 (0.72-7.61)	.16	0.79 (0.31-1.99)	.62	-	-
BTR strategy	0.91 (0.20-4.10)	.90	0.44 (0.16-1.22)	.11	5.95 (1.59-22.29)	.008

*BSA,* Body surface area; *ECMO,* extracorporeal membrane oxygenation; *CHD,* congenital heart disease.

## Supplementary Data

Video 1 Senior author explains the technique of Berlin Heart EXCOR ventricular assist device removal. Video available at: https://www.jtcvs.org/article/S2666-2736(25)00321-3/fulltext.
