# Supplementary figures and images for: Experience with Berlin heart support in children with a focus on device removal
Source: JTCVS Open. 2025 Sep 25;28:479–94. doi: 10.1016/j.xjon.2025.09.025 (PMC12745095; doi:10.1016/j.xjon.2025.09.025)

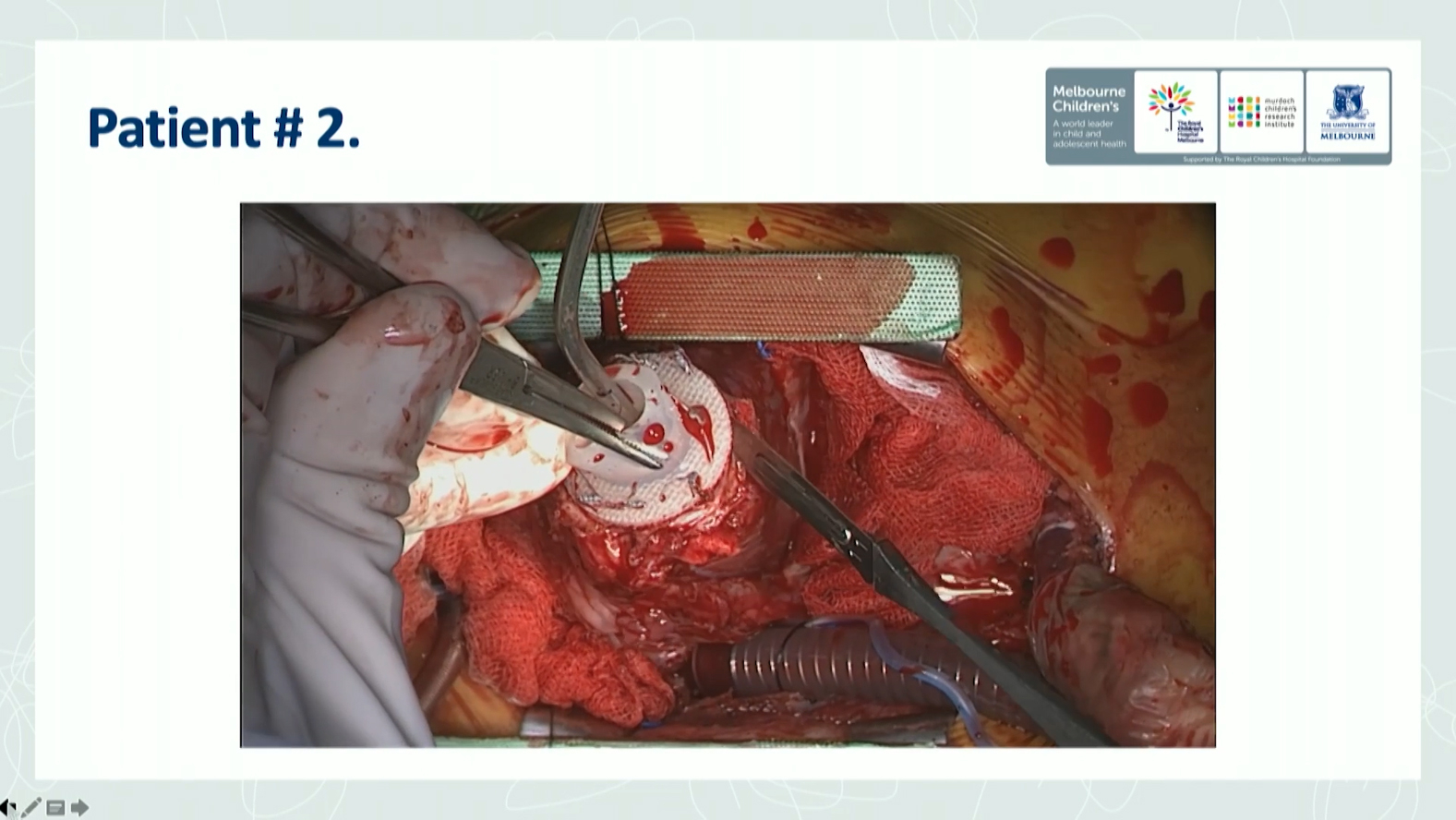

Supplement: Video 1 — Senior author explains the technique of Berlin Heart EXCOR ventricular assist device removal. Video available at: https://www.jtcvs.org/article/S2666-2736(25)00321-3/fulltext. [file fx2.jpg]
